# Sex Disparity Among Faculty of Physiology in North American Academia: Differences in Scholarly Productivity and Academic Rank

**DOI:** 10.7759/cureus.11850

**Published:** 2020-12-02

**Authors:** Imad Ahmad, Najib U Khan

**Affiliations:** 1 Medicine, Vancouver General Hospital, Vancouver, CAN; 2 Internal Medicine, Medical Unit, Abbasi Shaheed Hospital, Karachi, PAK

**Keywords:** sex disparity, sex differences, sex disaggregated data

## Abstract

Medical academic research done in various specialties shows sex disparity in terms of academic and leadership rank. Research shows that in many medical academic research fields, there are a greater number of men with higher academic and leadership ranks, as well as higher research productivity. This begs the question: What is the case for medical academic research specifically in physiology departments throughout North America? Upon review of the literature, we found that a knowledge gap still exists in North America regarding sex differences among the faculty of physiology. Our rationale for this study is that if a sex disparity among the faculty of physiology in North American academia is found, steps can be taken to lower this disparity. The very first step is identifying that a problem exists. Scopus was used to obtain the h-index, years of active research, and the number of publications and citations of each faculty member. The h-index was used as a metric of academic output and scholarly productivity. Univariate regression was run with the h-index as the outcome of interest and multiple linear regression analysis was used to determine factors associated with a higher h-index. The analysis showed that while the overall number of females holding academic positions in physiology departments throughout North America has increased over the years, a large sex disparity still exists between males and females in the field. This disparity exists not only in academic and leadership rank but also in research productivity, a key predictor of success in the field. This finding warrants that further work be done to find what is causing this disparity and how it can be addressed.

## Introduction

Medical academic research done in various specialties shows a sex disparity in terms of academic and leadership rank. Physiology is defined as the study of how biological systems act at the molecular, cellular, and organ system level, a definition which itself embodies not only the relevance of the field but also the extensive subspecialties that can exist in it. Evidently, it is not hard to justify the importance of the development of academic physiology, in that it would have strong implications for the development and progress of medical science as a whole. Given the importance and relevance of the specialization of physiology, as well as females matching, or even outperforming in some cases, males in certain academic and academic research circumstances, males and females should be at least equally represented within the field of physiology through academia, in terms of measures such as research productivity and academic rank [1-10].

A study done by Pell et al. showed that females were just as competent as their male counterparts in measures of research capabilities [8]. However, research shows that in many medical academic research fields, there are a greater number of males with higher academic and leadership ranks, as well as higher research productivity. This begs the question: what is the case for medical academic research specifically in physiology departments throughout North America?

Upon review of the literature, we found that a knowledge gap still exists in North America regarding sex differences among the faculty of physiology. Our rationale for this study is that if a sex disparity among the faculty of physiology in North American academia is found, steps can be taken to lower this disparity. The very first step is identifying that a problem exists. A field as impactful and relevant, as physiology should definitely have equal representation of both males and females in its academic research. In addition, a gap in the data exists because although there have been many studies published on sex disparity in academia across medical fields; there is currently no research specifically on physiology research [1-10].

## Materials and methods

We have examined the metric indices in the Elsevier database Scopus. Physiology faculty members’ names, academic ranks, leadership positions, and sex were obtained from each institutions’ website. Scopus was used to obtain the h-index, years of active research, number of publications, and citations of each faculty member. Faculty members from 63 different institutions offering physiology programs, located in North America, were included in our data set. For each of the faculty members surveyed at these institutions, we recorded their sex, academic rank, leadership rank, number of documents, h-index, citations, publication range, years since first publication, and years of active research from the institutional database and the database Scopus. The h-index was used as a metric of academic output and scholarly productivity. Univariate regression was run with the h-index as the outcome of interest and multiple linear regression analysis was used to determine factors associated with a higher h-index. Logistic regression was run to calculate the odds ratio. Data were tested for normality using the Kolmogorov Smirnoff test. Since the data were not normally distributed, we had analyzed, using median and range, for the quantitative variable. Frequency and percentages were calculated for the qualitative variable. The Pearson correlation test was applied. P-value <0.05 was considered significant. Binary logistic regression was used to calculate the odds ratio. STATA version 14.2 (StataCorp, College Station, Texas) was used for analysis.

## Results

**Figure 1:**
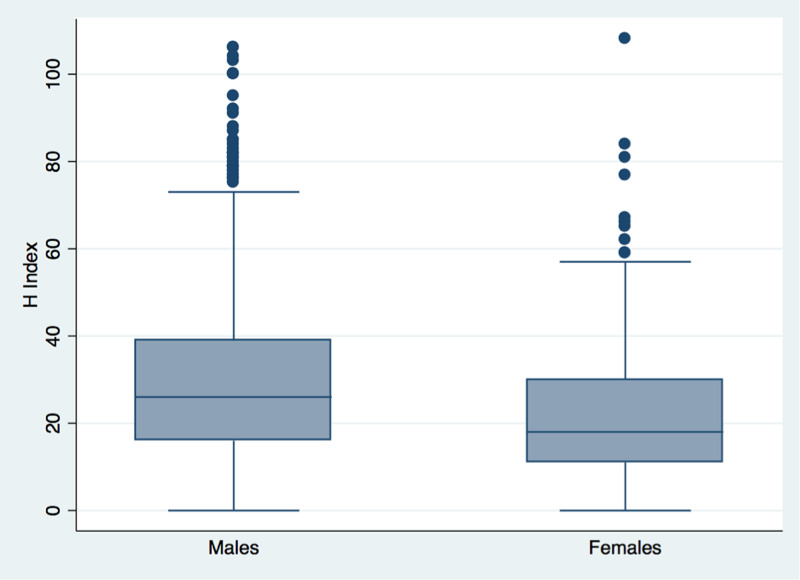
Distribution of h-index across males and females

In our sample size, there were a total of 1860 faculty members whose sex we were able to identify, out of which 1374 (73.8%) were males and 486 (26.13%) were females. Overall, 1554 (83.55%) were from the USA and 306 (16.45%) were from Canada. There were 1144 (83.26%) males in USA, 230 (16.74%) in Canada. There were 410 (84.36%) females in the USA and 76 (15.64%) females in Canada. The distribution of male and female faculty in physiology was the same in both the USA and Canada (chi-square 0.317, p-value = 0.573). The highest number of males was found in Ontario (120, 8.81%), New York (104, 7.63%), and Washington (85, 6.24%), and the least number of males was found in Oregon (3, 0.22%), Utah (9, 0.66%), and Oklahoma (9, 0.66%). The highest number of females was found in Ontario (32, 6.64%), Washington (36, 7.47%), and New York (45, 9.33%), and the least number of females was found in Kansas (2, 0.41%), Indiana (3, 0.62%), and Delaware (3, 0.62%). Males were in a clear majority in all states and provinces, with Rhode Island being the only exception (7 males and 10 females). Figure 1 shows the distribution of the h-index across males and females.

**Figure 2:**
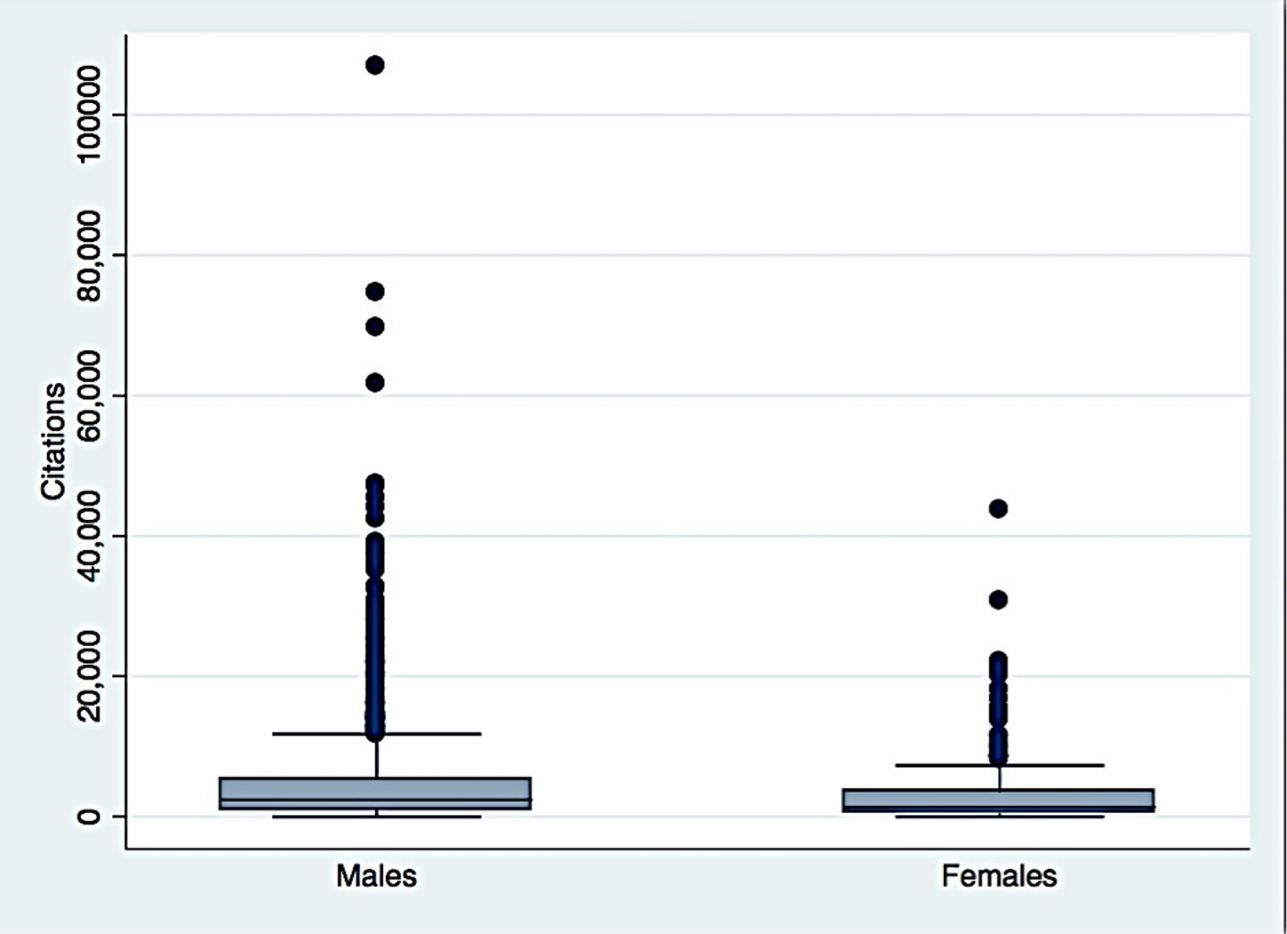
Distribution of citations across males and females

Figure 2 shows the distribution of citations across males and females.

We had checked the distribution of the h-index, publications, citations, and years of research across both sexes. Males had higher h-index, citations, publications, and more years of research than females.

We checked for the correlation between continuous variables. The variables citations and publications were highly correlated ( rho=0.75; p-value <0.0001). We included publications in the regression model and dropped citations.

Table 1 shows the distributions of publication, h-index, citations, and years of active research in relation to sex and academic range.

**Table 1:** Distribution of publications, citations, h-index, and years of research across the male and female faculty of physiology

Academic Rank	Male (freq)	Median (range)	Female (freq)	Median (range)
Publications				
Professor	819	100 (1-1132)	194	77.5 (1 -366)
Associate Professor	297	43 (1 - 308)	121	38 (1 - 147)
Assistant Professor	236	23 (1 - 265)	155	18 (1 - 82)
Citations				
Professor	811	4057 (0 - 106794)	192	3355 (0 - 43833)
Associate Professor	295	1536 (0 - 20098)	118	1254.5 (1 - 6724)
Assistant Professor	230	668.5 (1 - 12494)	145	538 (0 - 14153)
h-index				
Professor	810	34 (0 - 110)	193	30 (0 - 108)
Associate Professor	295	20 (0 - 72)	118	18.5 (1 - 42)
Assistant Professor	230	14 (1-64)	145	11 (1 - 38)
Years of Research				
Professor	813	30 (0 - 62)	194	31 (0 - 61)
Associate Professor	297	21 (0 - 54)	118	21 (0 - 43)
Assistant Professor	231	14 (0 - 42)	146	13 (0 - 31)

Table 2 checks for the correlation between continuous variables.

**Table 2:** Checking for the correlation between continuous variables

	Publications	h Index	Citations	Years of research
Publications	1.0			
H Index	0.0411 (p value=0.07)	1.0		
Citations	0.7502 (p value<0.0001)	0.0 (p value = 0.71)	1.0	
Years of research	0.5498 (p value<0.0001)	0.0226 (p value = 0.33)	0.4215 (p value<0.0001)	1.0

**Figure 3:**
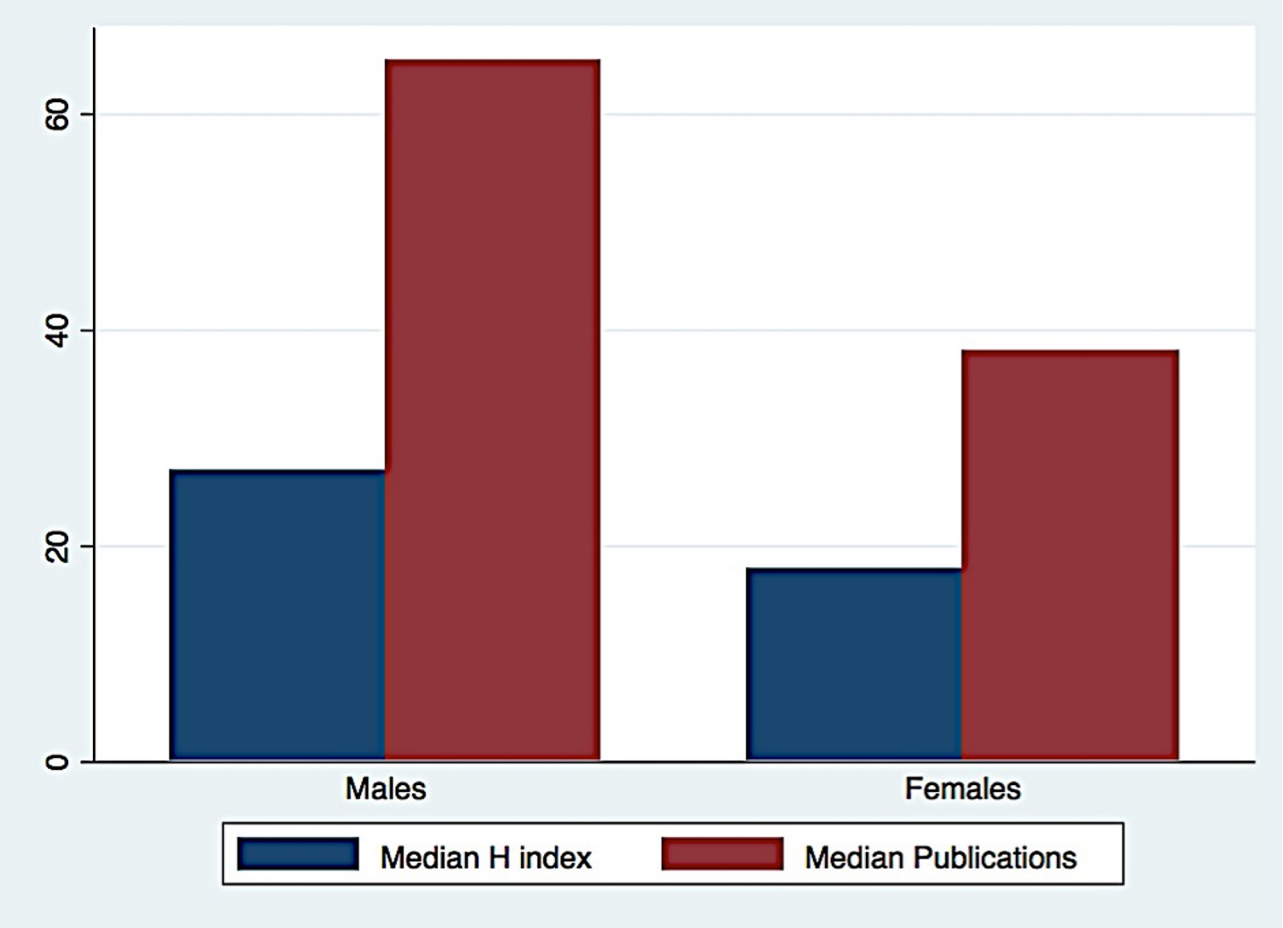
Distribution of median h-index and publications for males and females

Figure 3 shows the distribution of median h-index and publications for males and females. 

A Mann-Whitney U-test was used to test for the difference between males and females. It showed that there was a significant statistical difference between males and females in the categories of publications, citations, h-index, and years of active research, in which males had a greater number in each of these categories.

Since there were more than two categories in the academic rank, we had used a Kruskal-Wallis test. There was a significant statistical difference between males and females across academic ranks in the categories of publications, citations, h-index, and years of active research, in which males had a higher number in each of these categories.

We ran a univariate regression with h-index as the dependent variable. Significance was found for the variable of sex (p-value = 0.04). Median h-index differed between males and females, and males had a higher median h-index (p-value = 0.03). On average, males had a higher number of publications (p-value = 0.04). Median years of active research was higher for females (p-value = 0.04). The leadership variable was not significant and was removed from further analysis.

Median h-index of academic ranks was greater for males across professor level (p-value = 0.04), associate professor level (p-value = 0.01), and assistant professor level (p-value = 0.02) titles. However, female professors had a higher median h-index (p-value = 0.002).

Based on the results of the univariate regression, the variables that were taken forward in the multivariable analysis based on the cut-off value of 0.25 were as follows: sex (p-value=0.049); publications (p-value = 0.03); years of active research (p-value = 0.02); academic rank (p-value = 0.03). Main effects were identified using a stepwise selection strategy and based on the p-values. Variables with a level of significance (p value<0.05) were included in the model.

The final step was to check for interaction (cut-off p-value = 0.1). Interaction terms were created between each of the independent variables in the model, sex, publications, academic rank, and years of research. There were no significant interactions noted.

The prediction equation used accounted for major variability in our final model, which was demonstrated by an adjusted R square = 0.61 and p-value = 0.03. This shows that 61% of the variability in this model is explained by this model.

Running a binary logistic regression with sex as the dependent variable, we found that males had higher odds of having a higher h-index than females while adjusting for other covariates.

## Discussion

Many fields within academic medicine have been shown to contain a sex disparity over the past several years [8,10-11]. Although the number of female physicians has increased dramatically over the past years, this does not necessarily lead to a decrease in sex imbalance among physicians who hold senior academic ranks and leadership positions [12-16]. Our study found that females were in a significantly lower number than males. Females had a significantly lower number of publications than males and a lower h-index.

In the current study, research productivity was quantified by the h-index. The h-index is an important quantitative factor that contributes to career advancement and promotion [14-15]. The findings of this study revealed that women perhaps need more mentoring so that they have more opportunities to publish more quality research papers. Other factors may be contributing to the sex gap present in academic and leadership positions. These same results have been seen in a number of papers looking at various medical fields such as neurosurgery, academic interventional radiology, physical medicine, and rehabilitation [17-20]. These papers were also using the h-index as their variable to quantify research productivity.

This means that it is true that the increase in the number of female physicians in all fields does not lead to a decrease in sex disparity. One of the more popular opinions on what causes this disparity is sex-related myths and bias [17]. Because our results show that not only were there fewer women in the field of academic physiology, but the women who were in academic physiology had a lower h-index, our variable for research productivity; the problem is not only that fewer women tend to go in this field. A problem exists internally within the department, causing women to either have less ambition to strive for higher research productivity, or for others who are working and hiring in the field, to allow bias to influence their decisions about hiring women and giving them academic opportunities such as publishing.

Although there has not been a large amount of progress over recent years, past events have shown us the importance of women in academic physiology research. Among 1913 women, six were given membership to The Physiological Society, and each of them proved to be a valuable asset to the team [21].

Several factors may contribute to the promotional success of females. This disparity can exist for a large number of reasons. Marital status and number of children (or whether or not the woman has children at all) may be factors influencing the disparity. These are variables that would require further study to determine their influence on things like research productivity.

Regarding lifestyle choices and family responsibilities, differences have been well-documented. Literature has shown that in the past [22] and in present times [23], females are found to be more likely to work part-time. More females have interruptions in academic training, with the biggest gap in academic continuity during pregnancy [24]. It is shown that females reported that family responsibility negatively affected their careers [25]. 

As years of active research are determined by an author’s year of first publication, time away for family or maternity leave is not accounted for, a finding that may disproportionately affect females who have their years of active research overestimated. Similarly, institutional or international changes during one’s years of active research are unknown. Pertaining to leadership positions, no distinctions were made to the hierarchy within leadership positions. At some institutions, leadership positions are held on a rotation or fixed-term basis. Data for progression through academic rank from the time of initial appointment is not known.

As both the actual trajectory and expected trajectory of an individual’s academic career path are challenging to characterize, the disparity in career advancement through job promotion and having more publications are likely multifactorial. Programs should address sex disparities within their physiology departments, with a long-term goal of enhancing sex equality.

Limitations

This study has several limitations. Elsevier’s Scopus database was used to collect the data regarding the scholarly performance of the physiology faculty. All metrics in Scopus are quantitative. One limitation is that while in basic sciences, the quality of studies and research is important and not just their quantity, we did not have access to those data that would enable us to gauge the quality of their research work. Hence, this issue has been overlooked in this study. For the purpose of this study, there were several assumptions that we had made. We had abided by the assumption that physiology faculty who are absent from Scopus have no published papers. This assumption may be flawed. Credit for published papers could perhaps be misplaced due to a change in the author's name after a marriage or divorce. This would affect females more than males. This again is a bias. Furthermore, our sampling may be subject to bias, as the information on departmental websites may be old. Maybe the websites have not been updated. The h-index is not an ideal measure and does not differentiate between the order of authorship, does not differentiate between several papers of substandard quality and one paper of good quality, nor does it take into account the number of self-citations. Years of active research are calculated by an author’s year of first publication, time away for the family, or maternity leave is not accounted for. This is a finding that may disproportionately affect females who have their years of active research overestimated. Data for progression through academic rank from the time of first clinical appointment is unknown. Sex differences breakdown with the percentage of time served as a full-time faculty versus part-time employment, and contract versus tenure positions is also not known. All these are the limitations to our study that we had thought about while conducting this study. We are aware that there may be more limitations.

## Conclusions

While the overall number of females holding academic positions in physiology departments throughout North America has increased over the years, a large sex disparity still exists between males and females in the field. This disparity exists not only in academic and leadership rank but also in research productivity, a key predictor of success in the field. This finding warrants that further work be done to find what is causing this disparity, and how it can be addressed.
